# Transcriptional activator DOT1L putatively regulates human embryonic stem cell differentiation into the cardiac lineage

**DOI:** 10.1186/s13287-018-0810-8

**Published:** 2018-04-10

**Authors:** Varsha Pursani, Deepa Bhartiya, Vivek Tanavde, Mohsin Bashir, Prabha Sampath

**Affiliations:** 1 Stem Cell Biology Department, ICMR-National Institute for Research in Reproductive Health, J.M. Street, Parel, Mumbai, Maharashtra 400 012 India; 2 Division of Biological & Life Sciences, School of Arts & Sciences, Ahmedabad University, Ahmedabad, 380009 India; 3 Genome and Gene Expression Data Analysis Division, A* Star—Bioinformatics Institute, Singapore, 138671 Singapore; 4 Division of Translational Control of Disease, A* Star—Institute of Medical Biology, Singapore, 138648 Singapore

**Keywords:** Human embryonic stem cells, Cardiac differentiation, DOT1L, Epigenetics, Gene expression, Histone methyltransferase

## Abstract

**Background:**

Commitment of pluripotent stem cells into differentiated cells and associated gene expression necessitate specific epigenetic mechanisms that modify the DNA and corresponding histone proteins to render the chromatin in an open or closed state. This in turn dictates the associated genetic machinery, including transcription factors, acknowledging the cellular signals provided. Activating histone methyltransferases represent crucial enzymes in the epigenetic machinery that cause transcription initiation by delivering the methyl mark on histone proteins. A number of studies have evidenced the vital role of one such histone modifier, DOT1L, in transcriptional regulation. Involvement of DOT1L in differentiating pluripotent human embryonic stem (hES) cells into the cardiac lineage has not yet been investigated.

**Methods:**

The study was conducted on in-house derived (KIND1) and commercially available (HES3) human embryonic stem cell lines. Chromatin immunoprecipitation (ChIP) was performed followed by sequencing to uncover the cardiac genes harboring the DOT1L specific mark H3K79me2. Following this, dual immunofluorescence was employed to show the DOT1L co-occupancy along with the cardiac progenitor specific marker. DOT1L was knocked down by siRNA to further confirm its role during cardiac differentiation.

**Results:**

ChIP sequencing revealed a significant number of peaks characterizing H3K79me2 occupancy in the proximity of the transcription start site. This included genes like *MYOF*, *NR2F2*, *NKX2.5*, and *HAND1* in cardiac progenitors and cardiomyocytes, and *POU5F1* and *NANOG* in pluripotent hES cells. Consistent with this observation, we also show that DOT1L co-localizes with the master cardiac transcription factor *NKX2.5*, suggesting its direct involvement during gene activation. Knockdown of DOT1L did not alter the pluripotency of hES cells, but it led to the disruption of cardiac differentiation observed morphologically as well as at transcript and protein levels.

**Conclusions:**

Collectively, our data suggests the crucial role of H3K79me2 methyltransferase DOT1L for activation of NKX2.5 during the cardiac differentiation of hES cells.

**Electronic supplementary material:**

The online version of this article (10.1186/s13287-018-0810-8) contains supplementary material, which is available to authorized users.

## Background

Pluripotent stem cells (PSCs) are blank cells with the ability to differentiate into multiple cell types depending upon the cues provided *in vitro*. They have open euchromatin and complex epigenetic changes occur when these PSCs become committed. Among these, histone modifications take up the major role of opening the chromatin structure for the subsequent transcription activation. A number of studies have started unlocking the molecular mechanisms of these epigenetic factors that precisely orchestrate the development of specific cell types from undifferentiated PSCs to aid in their wide applications. Bivalency PSCs is a central discovery involving an interesting interplay of histone methylations H3K27me3 and H3K4me3. Deposited by EZH2 of the polycomb group (PcG) and MLL2 of the trithorax group (TrxG) of proteins respectively, bivalent domains are the most widely studied mechanisms that render the gene inactive and active while, on the other hand, the presence of both marks keeps the gene poised for subsequent activation or suppression upon differentiation [1–6].

The relative distribution of these bivalent marks has been extensively uncovered, assigning them a crucial role in various mammalian developmental processes including cardiogenesis. Our group recently reported a vital role for EZH2 in the cardiac differentiation process wherein EZH2 is recruited by NR2F2 (cardiac marker) at the OCT4A promoter (pluripotency marker) for its repression in early cardiac differentiation stages by bringing about an H3K27me3 mark [7]. In addition to MLL2, there are other histone active methyltransferases recruited at the gene to activate transcription by methylating the target locus.

Histone methyltransferases have been shown to be guided at the genomic locations in specific cell types by directive roles of signaling pathways, histone variants, nucleosome remodeling, and transcription factors [8–11]), although the mechanistic and specificity details are still left to be uncovered. Cardiac differentiation has also been shown as an integration of genomic (transcription factors) and epigenetic (histone methyltransferases) information that collectively activates and deactivates the cardiac specific machinery. Epigenetic connection of cardiac formation was first put forward in 2005 when the key transcription factor GATA4 was shown to be coactivated by an acetylation mark brought about by histone acetyltransferase p300, thereby increasing its DNA binding ability and stability in cardiac myocytes differentiated from ES cells [12]. Activated GATA4 further binds to NKX2.5, another master cardiac transcription factor (TF) triggering cardiogenesis [13, 14]. Similarly, essential roles for histone demethylases like UTX and JMJD3 (H3K27me3 demethylases) have been reported to activate the cardiac genes during ES cell transition from pluripotency to cardiomyocytes [15–17]. NKX2.5 functions as an instrumental part of each of the differentiation stages like chamber formation, patterning of the conduction system, formation of the interventricular septum, defined expression of critical downstream genes, and terminal differentiation of the myocardium followed by their maturation into adult equivalents [18–22]. Understanding the signals and the modifications for the expression of cardiac transcription factors thus remains necessary to expose the mechanistic details for stepwise depiction of cardiac development.

DOT1L, unlike all other histone methyltransferases, represents the first crucial histone methyltransferase not containing an evolutionarily conserved catalytic domain called SET, referring to the Su(var)3-9, Enhancer of Zeste (E(Z)), and Trithorax (trx) domain [23]. DOT1L represents the only enzyme that activates its target by delivering dimethylation at lysine 79 of histone H3 [23–28]. Although DOT1L was initially identified for regulating heterochromatin formation [29, 30], accumulating literature now suggests its role in the regulation of gene activation [31–34]. An important area having DOT1L as an essential controller is that of cell cycle and DNA damage repair [35–37]. Involvement of DOT1L in *in-vivo* cardiac development and function has been shown by Nguyen and Zhang [38], wherein the group noted severe dilated cardiomyopathy in DOT1L knockout mice, which upon further study was rescued by ectopic expression of DOT1L, and that DOT1L is the possible target malfunctioning in dilated cardiomyopathy. The contribution of DOT1L in cardiac formation from undifferentiated mouse ES cells was reported recently [39]. The study successfully proved DOT1L expression on cardiac genes, which upon knocking down affects the expression of these genes delaying the cardiac differentiation. To conclude, DOT1L has an important role during cardiogenesis both *in vivo* and *in vitro,* and demands much more research efforts toward displaying its connection at the molecular and genetic levels as its deletion results in cardiac pathogenesis.

The present study was designed to understand whether DOT1L is crucial for the cardiac progenitor differentiation *in vitro*. Studies were carried out on the inhouse-derived hES cell line KIND1 along with a well-studied HES3 hES cell line. By performing chromatin immunoprecipitation (ChIP) followed by sequencing (ChIP-seq), we interrogated the hES cell-derived cardiac progenitors and beating CMs for the occupancy of an H3K79me2 mark on the specific cardiac genes. Dual immunofluorescence was performed to investigate whether cardiac specific transcription factor NKX2.5 is coexpressed with and activated by H3K79me2 methyltransferase DOT1L.

## Methods

### Cell culture and differentiation

KIND1 is an in-house derived hES cell line derived at our laboratory in Mumbai [40] and the HES3 cell line (WiCell Research Institute Inc.) was available from Dr Prabha Sampath’s laboratory for the present study.

Undifferentiated feeder-free KIND1 hES cells were cultured in Stempro hES SFM medium (Invitrogen, Carlsbad, CA, USA) supplemented with 8 ng of bFGF (Peprotech, NJ, USA) as described earlier [7], while the HES3 cell line was grown in mTeSR™1 medium (STEMCELL Technologies Inc., Canada) at 37 °C and 5% CO_2_. For subjecting the confluent pluripotent KIND1 and HES3 hES cells to cardiac differentiation, they were transitioned from growth medium into RPMI 1640 containing 5% B-27 and 1% glutamax (basal medium), and the differentiation protocol was followed as reported by our group earlier [41]. In brief, cells were first exposed to basal medium supplemented with 100 ng/ml Activin A (Peprotech) and 5 ng/ml of bFGF (R&D Systems, MN, USA) for 24 h. This was followed by 15 ng/ml BMP4 (R&D Systems) and 5 ng/ml bFGF (R&D Systems) in basal medium for another 4 days. Finally, the cells were treated with WNT pathway blocker DKK1 (Peprotech) at 150 ng/ml concentration for the next 4 days. From day 9 onward, the cells were maintained in basal medium until day 20 wherein the media were changed on every alternate day.

### Quantitative polymerase chain reaction

Total RNA was extracted using an RNeasy Mini Kit (Qiagen, Germany) and quantified using an ND1000 Spectrophotometer (NanoDrop Technologies, Inc., DE, USA). Then 500 ng was subjected to reverse transcription for cDNA synthesis using SuperScript^®^ III Reverse Transcriptase (Thermo Fisher Scientific, MA, USA) as per the manufacturer’s instructions using a 7900HT Fast Real-Time PCR System (Thermo Fisher Scientific). Quantitative polymerase chain reaction (qPCR) was performed using SYBR Green chemistry (Thermo Fisher Scientific) on a 7900HT Fast Real-Time PCR System. The program applied for amplification included 25 °C for 10 min, 50 °C for 50 min, and 85 °C for 5 min. The fold change was determined by the 2^–ΔΔCt^ method and was expressed relative to that of an internal control, RPLPO. The expression level of each gene transcript is normalized to a value of 1.0 for undifferentiated cells. The error bars represent ±standard error of the mean (SEM). All results are an average of at least three biological replicates. The primers used are presented in Table 1.

**Table 1:** Primer sequences

Gene	Primer sequence 5′–3′
RPLPO	Forward: CAGATTGGCTACCCAACTGTT
	Reverse: GGGAAGGTGTAATCCGTCTCC
DOT1L	Forward: GAGACCTCCTTCGACCTGGT
	Reverse: CGACGCCATAGTGATGTTGC
MESP1	Forward: CTCTGTTGGAGACCTGGATG
	Reverse: CCTGCTTGCCTCAAAGTG
MEF2C	Forward: CGAGATACCCACAACACACG
	Reverse: TTCGTTCCGGTGATCCTC
NKX2.5	Forward: CCTCAACAGCTCCCTGACTCT
	Reverse: ATAATCGCCGCCACAAACTCTCC
ISL1	Forward: TGATGAAGCAACTCCAGCAG
	Reverse: GGACTGGCTACCATGCTGTT
CTNT	Forward: GGCAGCGGAAGAGGATGCTGAA
	Reverse: GAGGCACCAAGTTGGGCATGAACGAC

### ChIP sequencing

ChIP was performed as per our recent report [7]. Briefly, about 1–2 million KIND1 and HES3 hES cells each harvested at days 0, 12, and 20 were subjected to formaldehyde crosslinking and sonication (Bioruptor; Cosmo Bio Co. Ltd, Japan). Sonicated protein–DNA complexes (200–500 bp) were precipitated with 10 μg of anti-H3K79me2 antibody (Cell Signaling Technology, MA, USA) overnight at 4 °C. Post thorough washing and elution, the ChIPped samples were subjected to standard DNA extraction protocol employing phenol:chloroform:isoamyl alcohol as per the manufacturer’s instructions (Thermo Fisher Scientific). Extracted DNA samples were sent to the Genome Institute of Singapore (GIS), Singapore for sequencing on Illumina HiSeq2500 sequencer. The analysis of sequencing results obtained was performed at Sandor Life Sciences Pvt. Ltd (Hyderabad, India). The raw sequencing reads mapped with Humanhg19 were aligned using Bowtie (Galaxy tool) while peak calling was performed using MACS (Galaxy tool). Integrative Genome Viewer [42] was used for visualization of the resulting peaks.

### Dual-immunofluorescence

The standard immunofluorescence protocol was followed to study expression of DOT1L and NKX2.5. Cells were grown in chamber slides followed by their differentiation and fixation with 4% paraformaldehyde (PFA) (Sigma-Aldrich, MO, USA) at days 0, 12, and 20 for 15 min followed by permeabilization with 0.3% triton X-100 (Sigma-Aldrich). Blocking was performed using phosphate buffer saline (PBS) containing 5% BSA (Sigma Aldrich) plus 1% normal goat serum (Bangalore Genei, Bangalore, India) for 60 min at room temperature. Cells were then incubated at 4 °C overnight with primary antibodies against DOT1L (1:200) (Abcam) and NKX2.5 (1:200) (R&D Systems) diluted in blocking buffer. Later the cells were incubated in  appropriate secondary antibodies (Thermo Fisher Scientific) diluted in blocking buffer for 2 h at room temperature. Representatives images  were captured using a confocal microscope (Olympus FV1000).

### Knockdown experiments

A small interfering RNA (siRNA)-based transfection technique for knocking down was employed to study the expression of DOT1L in both KIND1 and HES3 cells at days 0, 12, and 20. siRNAs for DOT1L along with a nontarget siRNA pool (control), with the following target sequences, were procured from GE Dharmacon™. DOT1L siRNAs (LQ-014900-01-0010), (1) UCACUAUGGCGUCGAGAAA, (2) GCUAUGGAGAAUUACGUUU, (3) GCAGAAUCGUGUCCUCGAA, (4) AAGAGUGGAGGGAGCGAAU; and nontarget siRNA pool (D-001810-10-20), (1) UGGUUUACAUGUCGACUAA, (2) UGGUUUACAUGUUGUGUGA, (3) UGGUUUACAUGUUUUCUGA, (4) UGGUUUACAUGUUUUCCUA. Lipofectamine™ RNAiMAX Transfection Reagent (Thermo Fisher Scientific) was used to dilute the siRNAs for transfection as per the manufacturer’s instructions. The pool of DOT1L siRNAs 1 and 3 gave the maximum knockdown at a concentration of 25 nM. Post incubation for 5 min, siRNA DOT1L–lipid complex diluted in Opti-MEM™ Reduced Serum Medium (Thermo Fisher Scientific) was added to the cells. The medium was changed with Stempro hES SFM growth medium after 24 h. Cells were harvested 72 h post knock down for subsequent analysis. For knocking down DOT1L in cardiac progenitors (day 12), transfection was performed at day 9 of differentiation protocol (described earlier), following which the progenitor cells were collected after 72 h (i.e., at day 12). Similarly, for knocking down the DOT1L at day 20 (cardiomyocyte stage),  siRNA transfections were performed at day 17 of the cardiac-directed differentiation protocol.

## Results

### Differentiation of hES cells: KIND1 and HES3 cells display an identical cardiac differentiation pattern

Sequential addition of growth factors to undifferentiated KIND1 and HES3 cells led to a stepwise differentiation pattern into the cardiac lineage. Directed differentiation of pluripotent HES3 cells into the cardiac lineage was associated with distinct morphological changes ( see Additional file 1 for images taken at different time points of differentiation). Similar changes were observed when KIND1 cells were subjected to a similar differentiation protocol recently reported by our group [7]. Activation of the BMP and TGF-β pathways with a high concentration of growth factor ligands like BMP4 and Activin A resulted in upregulation of transcripts like *GATA4* and *KDR2* that represent the formation of early cardiac mesoderm. Following this, addition of DKK1 for inhibition of the WNT pathway further drove the differentiation toward the cardiac fate evident by expression of *MESP1*, *MEF2C*, *ISL1*, *NKX2.5*, and *CTNT* transcripts specific for cardiac mesoderm, cardiac progenitors, and beating cardiomyocytes (observed only in KIND1 cells). Depending upon the gene expression pattern, we harvested the cells at days 0, 12, and 20 during differentiation of both KIND1 and HES3 cells, which depict undifferentiated pluripotent hES cells, cardiac progenitors, and beating cardiomyocytes for carrying out further studies. The corresponding changes in specific transcripts that were expected to change during differentiation and protein expression of *CTNT* and *NKX2.5* in differentiating HES3 cells are presented in Additional files 2 and 3. These results are also similar to earlier published data using KIND1 cells [7].

Interestingly with these results, we for the first time report the typical cardiac differentiation pattern following a 20-day directed differentiation protocol in the HES3 cell line. In addition, a similar morphology observed in both cell lines at each stage during cardiac differentiation, further validating our protocol as well as the pattern of cardiac differentiation from hES cells. Beating cardiomyocytes were observed only in KIND1 cells perhaps because of intrinsic differences between the two cell lines. KIND1 cells were derived on human feeder fibroblasts whereas HES3 cells were derived initially on mouse embryonic fibroblast support.

### ChIP-seq: H3K79me2 targets cardiac lineage genes during differentiation of KIND1 and HES3 cells

**Fig. 1:**
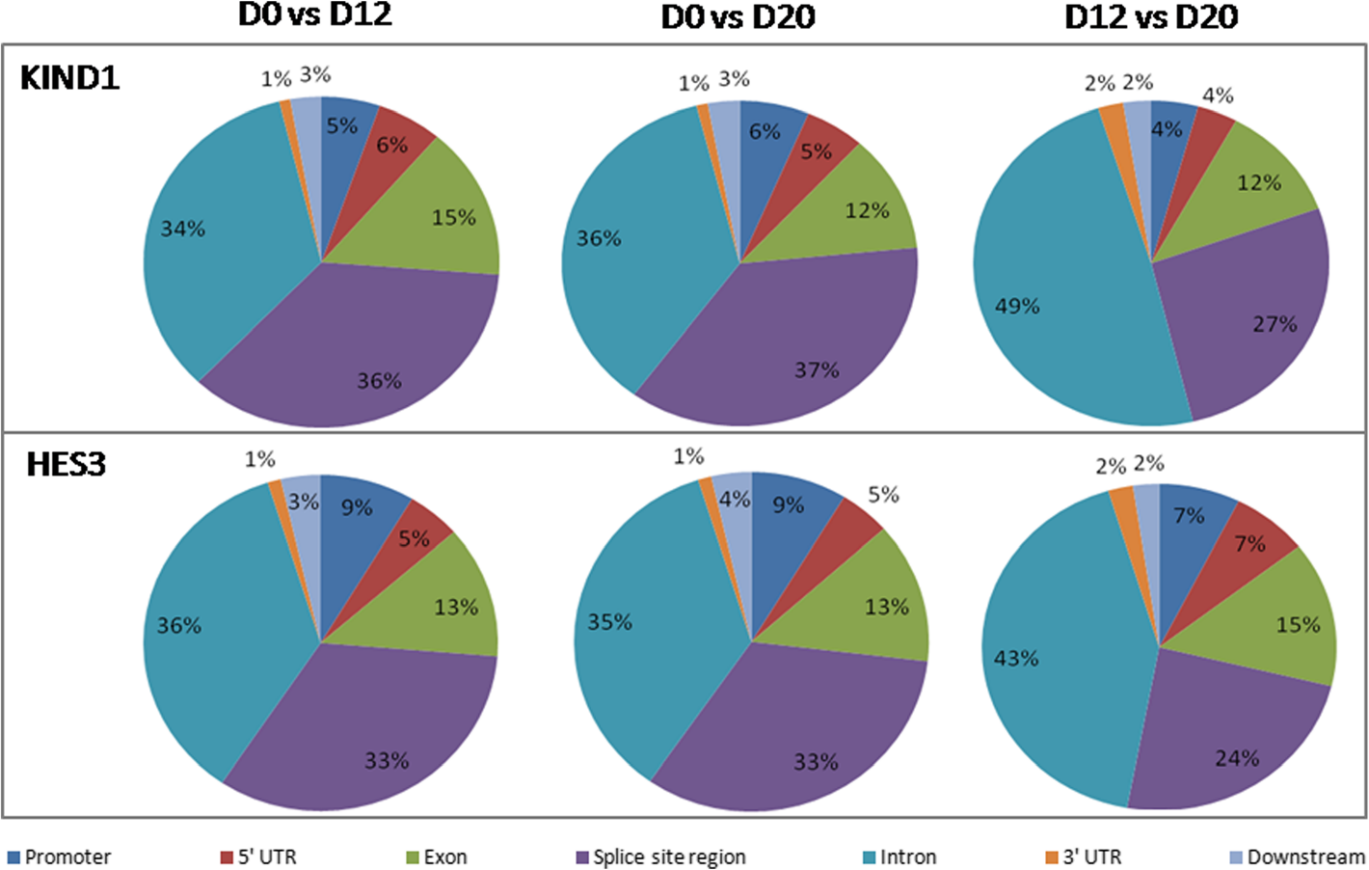
H3K79me2 peak deposition on genomic regions in (**a**) KIND1 and (**b**) HES3 cells. Pie charts represent disposition of H3K79me2 methyl mark over genomic regions, represented as percentage of peaks of KIND1 and HES3 cells during their directed differentiation into cardiac lineage from D0 to D12, D0 to D20, and D12 to D20. D day, UTR untranslated region

**Fig. 2:**
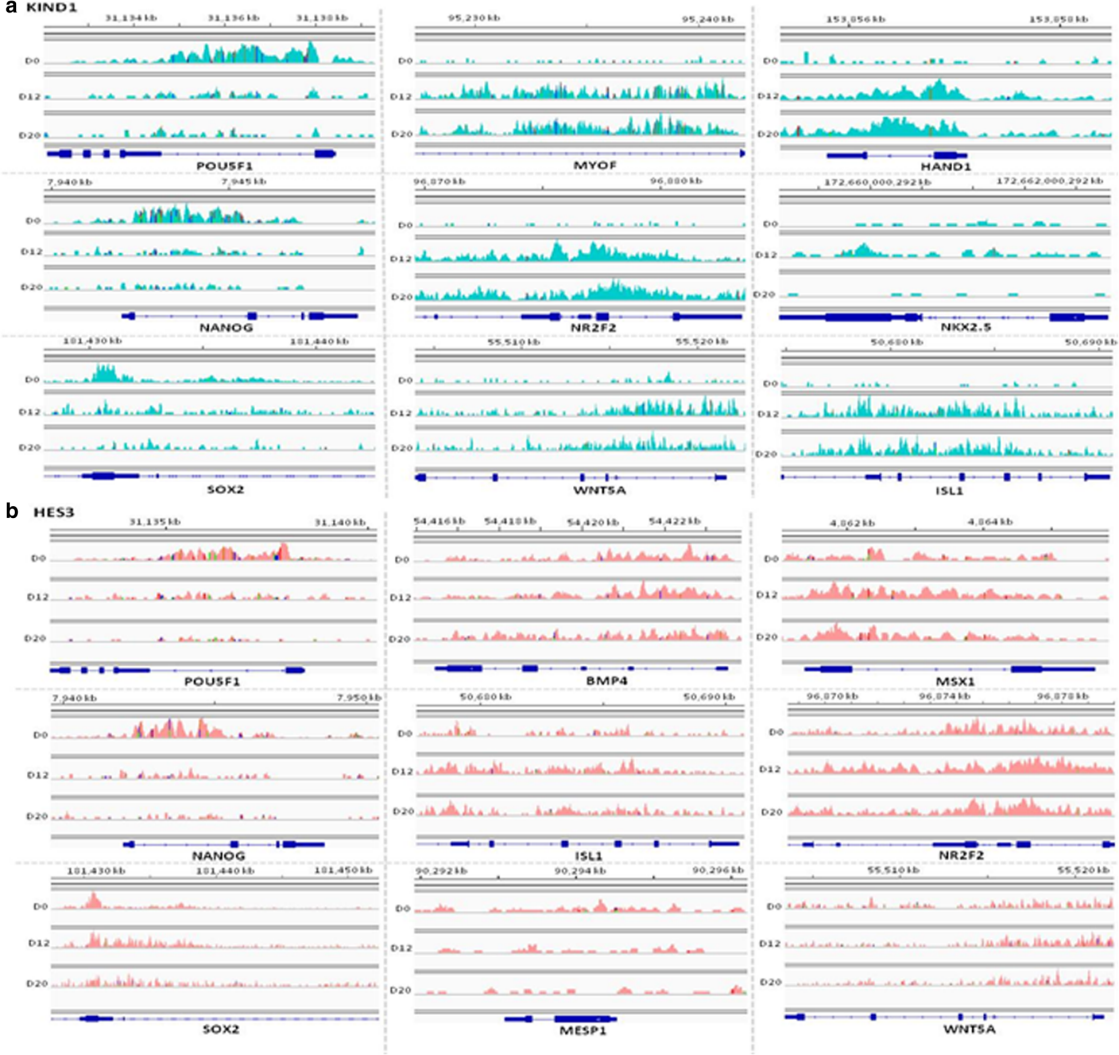
ChIP sequencing analysis in (**a**) KIND1 and (**b**) HES3 cells during differentiation. ChIP sequencing analysis visualized by integrated genome viewer shows binding profile of H3K79me2 modification across genes specific for pluripotency (*OCT4*, *NANOG*, *SOX2*); and mesodermal cardiac lineage (*MYOF*, *HAND1*, *NKX2.5*, *NR2F2*, *WNT5A*, *ISL1*) in KIND1 cells and (*BMP4*, *MSX1*, *ISL1*, *WNT5A*, *NR2F2*, *MESP1*) in HES3 cells at mentioned time points of cardiac differentiation

We first sought to understand the occupancy of H3K79me2 modification onto the genomic regions in undifferentiated as well as differentiating hES cells toward the cardiac lineage. Chromatin immunoprecipitation followed by sequencing (ChIP-seq) visualized an interesting expression pattern of an H3K79me2 methyl mark onto the expressed genes. The enrichment of H3K79me2 mark appeared upstream of exons including the promoters and the 5′UTR regions of genes, with a significant proportion of peaks occupying downstream regions of the transcription start site (TSS) as per the known localization pattern of this mark (Fig. 1). Analyzing the status of this dimethyl mark at day 0, transcripts like *POU5F1*, *NANOG*, and *SOX2* representing the pluripotent hES cells accumulated H3K79me2 peaks toward the downstream of TSS and disappeared at both the day 12 and day 20 stage of cardiac differentiation (Fig. 2). This highlights the possible role of an H3K79me2 methyl mark in their activation in an undifferentiated state. Further analyzing the cells at days 12 and 20, the active genes comprised key transcripts representing the cardiac lineage like *BMP4*, *GATA4*, *KDR2*, *ISL1*, *MEF2C*, *MSX1*, *MYH6*, *MYL2*, *NKX2.5*, *NR2F2*, *T*, *TNNT2*, *WNT5A*, *HAND1*, and *MYOF* (Fig. 2a, b). On extending our analysis, significant signals of H3K79me2 peaks were noted at the intron, 3′UTR, and splice site regions of genes including *HNF4A*, *LEFTY1*, *NOGGIN*, *NQO1*, *OTX2, SOX7, *and *NPTX2* (results shown for both KIND-1 and HES3 cells in Additional file 4). Expression patterns in both hES cell lines employed in the present study displayed high similarity, implying the crucial collaboration of H3K79me2 modification with the cardiac gene expression machinery. With respect to the various findings describing its roles in transcriptional activation or for the derepression of heterochromatin locally [31, 33, 43–45], DOT1L is also known as the critical regulator of the cell cycle process wherein enriched expression of DOT1L is found during the G2/M phase [36, 46].

On the other hand, deficiency of DOT1L results in cell cycle progression defects and aneuploidy in differentiating ES cells [35, 47]. In support of this, we in our present analysis also noted the significant peaks at *PCNA*, the gene required for DNA replication known as the marker of cell proliferation of cardiac muscle cells [48]. Additional cell cycle regulators containing an H3K79me2 mark include *BUB1* and * BRCA1* along with *E2F5*, an antiapoptotic factor in cardiomyocytes. These analyses, besides confirming the differentiation of KIND1 and HES3 hES cells into the cardiac lineage, also reveal the necessary involvement of dimethylation of H3K79 as a putative activation mark for induction of gene activation during cardiogenesis *in vitro*.

### Dual immunofluorescence: coexpression of DOT1L and NKX2.5 in cardiac progenitors

**Fig. 3:**
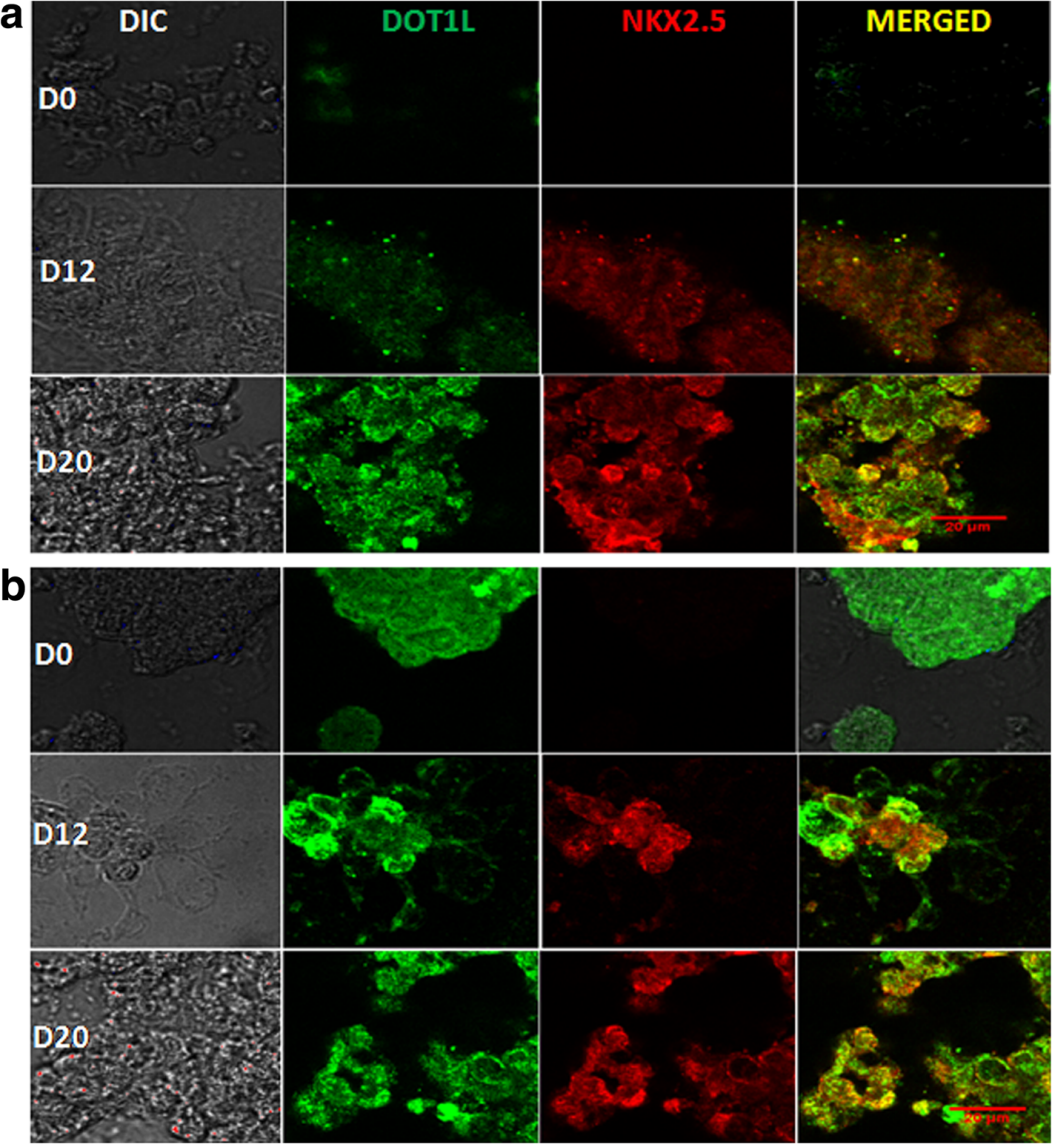
Coexpression of DOT1L and NKX2.5 in (**a**) KIND1 and (**b**) HES3 cells during differentiation. Dual immunofluorescence revealed coexpression of histone modifier DOT1L (green, nuclear) along with the master cardiac transcription factor NKX2.5 (red, nuclear) on days 12 and 20 during cardiac directed differentiation of KIND1 and HES3. These markers not expressed in undifferentiated cells (D0) of both KIND1 and HES3 cells. Scale bar: 20 μm. D day, DIC differential interference contrast

NKX2.5 is an essential marker for cardiac progenitors during *in-vivo* embryonic development as well as *in-vitro* differentiation of pluripotent cells. In continuation of the observation that an H3K79me2 mark occurs on NKX2.5, we were then interested to see whether H3K79me2 methyltransferase DOT1L is involved in activation of the NKX2.5 gene by delivering an H3K79me2 mark leading to its expression that is essential for the downstream cardiogenesis. This coappearance of DOT1L and NKX2.5 was investigated by dual immunofluorescence performed at days 0, 12, and 20. Figure 3 clearly reveals the significant number of areas coexpressing DOT1L and cardiac marker NKX2.5 at the progenitor (day 12) and the beating cardiomyocyte (day 20) stages obtained from KIND1 and HES3 cells. On the other hand, no colocalization was observed for DOT1L and NKX2.5 at the pluripotent (day 0) stage of the cells. These obvious results further indicate that DOT1L might be coactivating the key cardiac transcript NKX2.5 by bringing about an H3K79me2 activation mark.

In support of these results, Dystrophin (*DMD*) expression was upregulated in cardiac progenitors and beating cardiomyocytes compared to undifferentiated KIND1 cells (Additional file 5). ChIP sequencing analysis also revealed significant peaks representing the H3K79me2 occupancy at the *DMD* gene in differentiated cardiac cells obtained from both KIND1 and HES3 cells (see Additional file 6). In their mechanistic study, Nguyen and Zhang [38] revealed that DOT1L functions in cardiomyocytes through regulating *DMD* transcription and that *DMD* is a direct target of DOT1L. The present study clearly reports a correlated expression of *DMD* and DOT1L-mediated H3K79me2 methylation in cardiac cells. The results are in compliance with published studies and report the expression of *DMD* in cardiac cells differentiated from hES cells for the first time, further implying the active involvement of DOT1L during cardiac differentiation.

### Knockdown studies: DOT1L deficiency leads to compromised cardiogenesis from KIND1 and HES3 cells

**Fig. 4:**
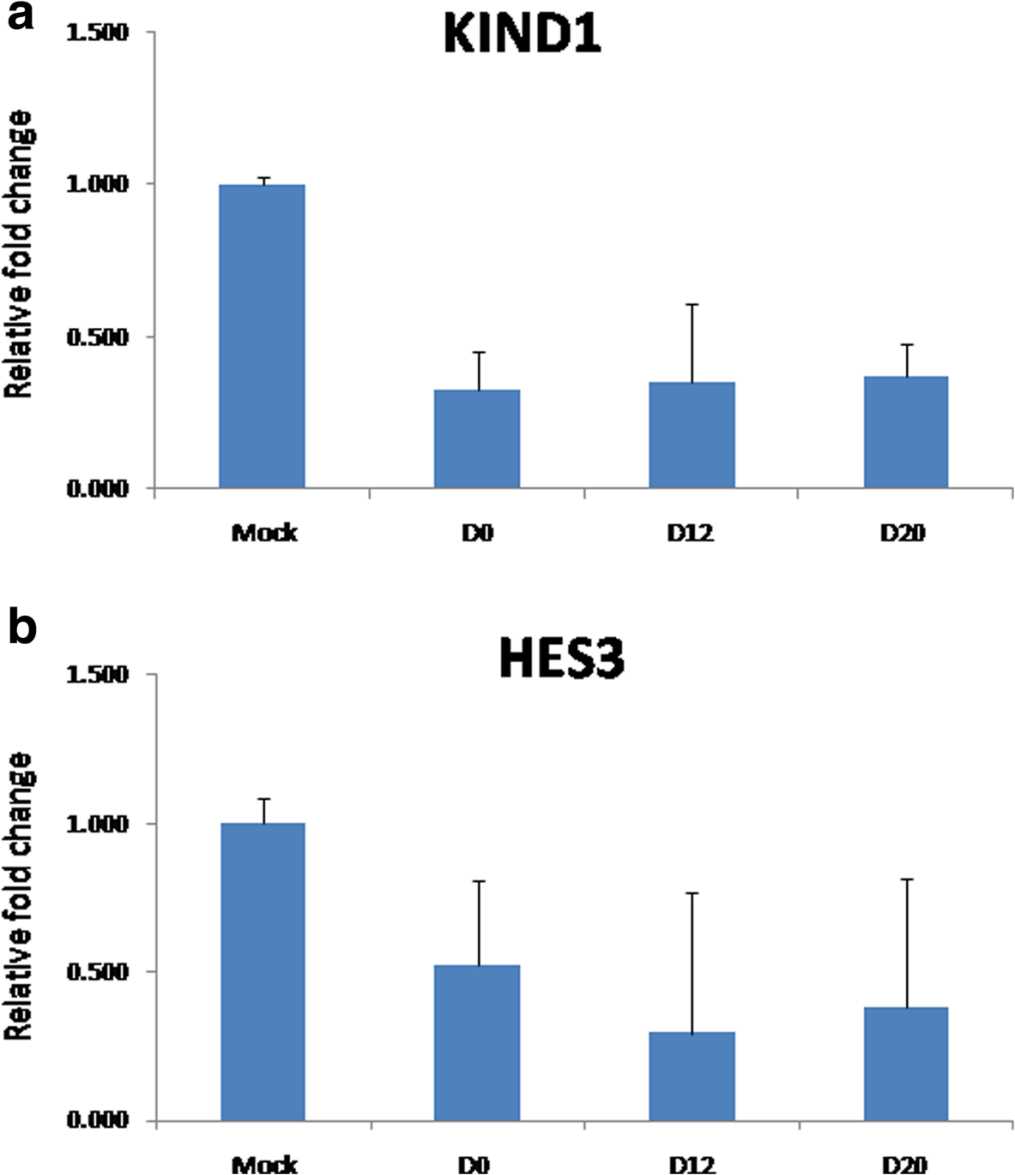
*DOT1L* expression in (**a**) KIND1 and (**b**) HES3 cells after DOT1L siRNA treatment. About 70–75% DOT1L knockdown was achieved by DOT1L siRNA treatment in both KIND1 and HES3 cell lines compared to mock control. Error bars represent ±SEM. Knockdown is a result of three independent experiments. D day

**Fig. 5:**
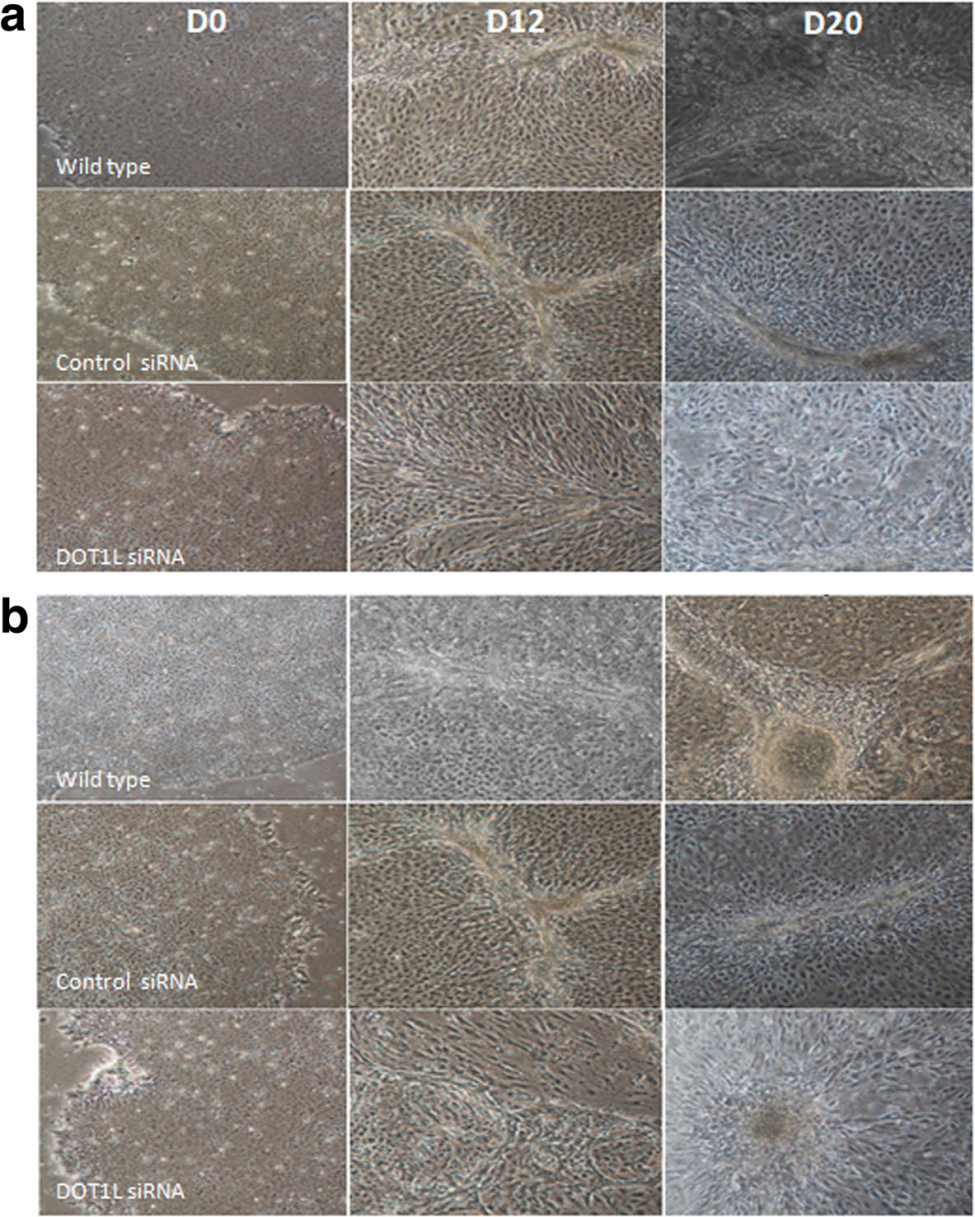
Effect of DOT1L knockdown on (**a**) KIND1 and (**b**) HES3 cell morphology on different days of differentiation. Both KIND1 and HES3 cells displayed mesenchymal morphology in their wild-type and nontarget siRNA-treated state characteristic of the mesodermal to cardiac differentiation as compared to the impaired cardiac differentiation pattern noted post DOT1L knockdown. Magnification 10×. D day

**Fig. 6:**
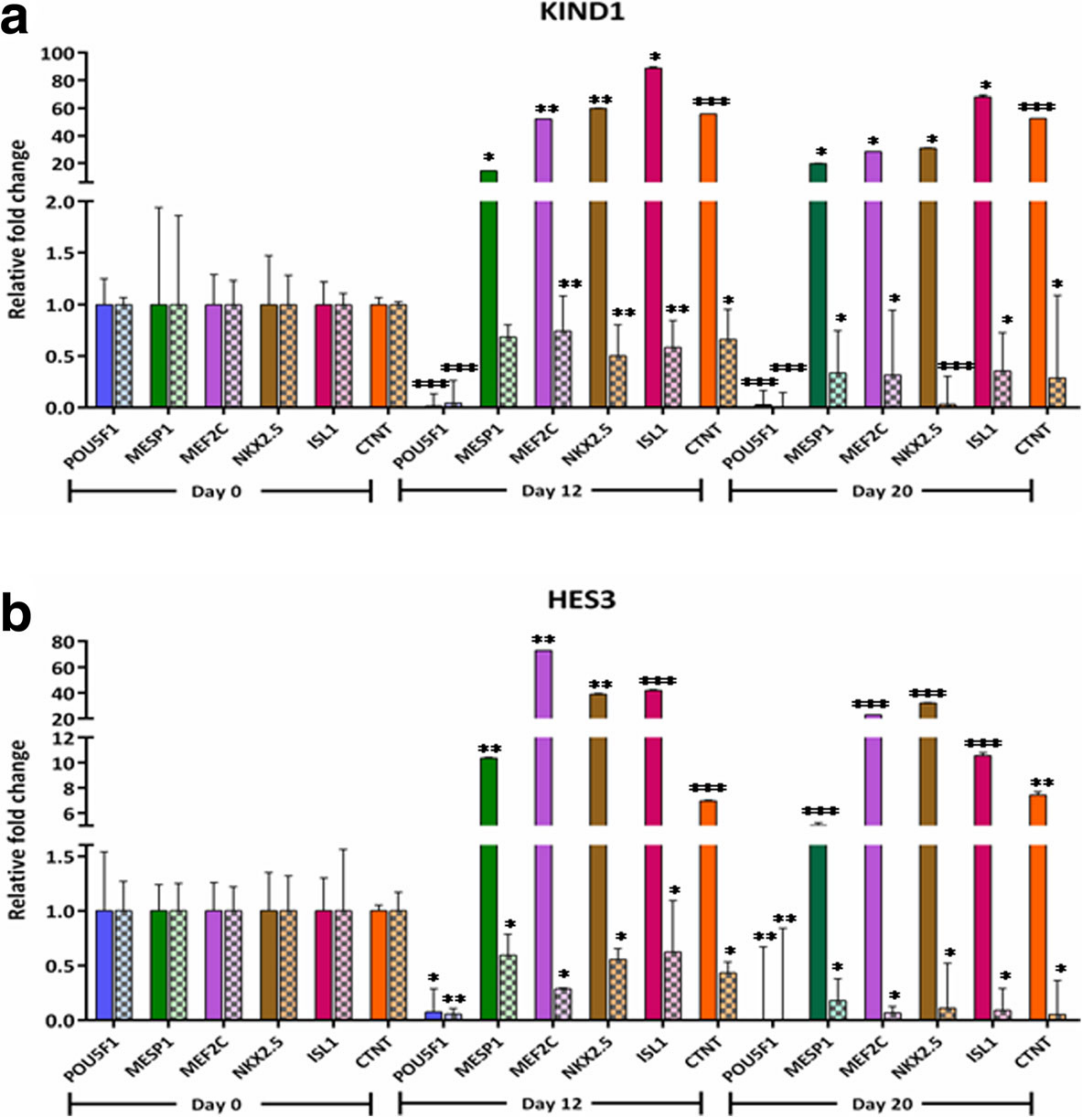
Effect of DOT1L knockdown on pluripotent and cardiac specific transcripts by qRT-PCR analysis in (**a**) KIND1 and (**b**) HES3 cells. Relative expression for pluripotency (*OCT4*) and cardiac specific (*MESP1*, *MEF2C*, *NKX2.5*, *ISL1*, *CTNT*) transcripts during differentiation of KIND1 and HES3 cells, normalized to internal control RPLPO and presented as fold change of expression. Nonpatterned bars represent expression levels in cells with nontarget siRNA while patterned bars represent expression in cells after DOT1L siRNA treatment. Error bars represent ±SEM of three independent knockdown experiments. Statistical significance: *p < 0.5, **p < 0.01, ***p < 0.001

Since DOT1L and its methylation mark H3K79me2 were found to be expressed along with NKX2.5, we then planned to explore whether DOT1L is crucially required in both regulating the pluripotent state as well as for obtaining cardiac progenitors. For this, we moved ahead with knocking down the DOT1L expression in both KIND1 and HES3 cells followed by their inspection both at pluripotent as well as during cardiac differentiation stages. In addition to comparing the gene expression status in DOT1L knocked down hES cells with that of wild-type hES cells, both cell lines were simultaneously transfected with nontarget siRNA control. With the achievement of about 70% and 75% of DOT1L knockdown in KIND1 and HES3 cells (Fig. 4), we first monitored its effect upon proliferation and maintenance of hES cells in their pluripotent state. Interestingly, post knock down both KIND1 and HES3 cells were morphologically indistinguishable when compared to their wild-type or control counterparts (Fig. 5). Confirming this at the transcript by qPCR, the expression of key pluripotent marker *OCT4* was seen to be unchanged in wild-type, negative control, and post-DOT1L knockdown hES cells at day 0 (undifferentiated) (Fig. 6). This is consistent with a couple of earlier reports stating that DOT1L does not inhibit the pluripotency of ES cells and that they retain the expression of pluripotent markers as evident at both transcript and protein levels [35, 39]. Extending ahead from the pluripotent state, we then worked further to examine the potential of both cell lines post knock down to differentiate and give rise to cardiac progenitors using the same differentiation protocol as described earlier. We first looked for the shift from the typical morphology that we observed during cardiac differentiation from KIND1 and HES3 cells as described. DOT1L knockdown severely impaired the normal differentiation of hES cell lines into cardiac progenitors, as evidenced by the depleted morphological pattern of cells at day 12 obtained post DOT1L knock down; as compared to that seen in cells containing no target siRNA pool or the wild-type KIND1 and HES3 cells (Fig. 5).

**Fig. 7:**
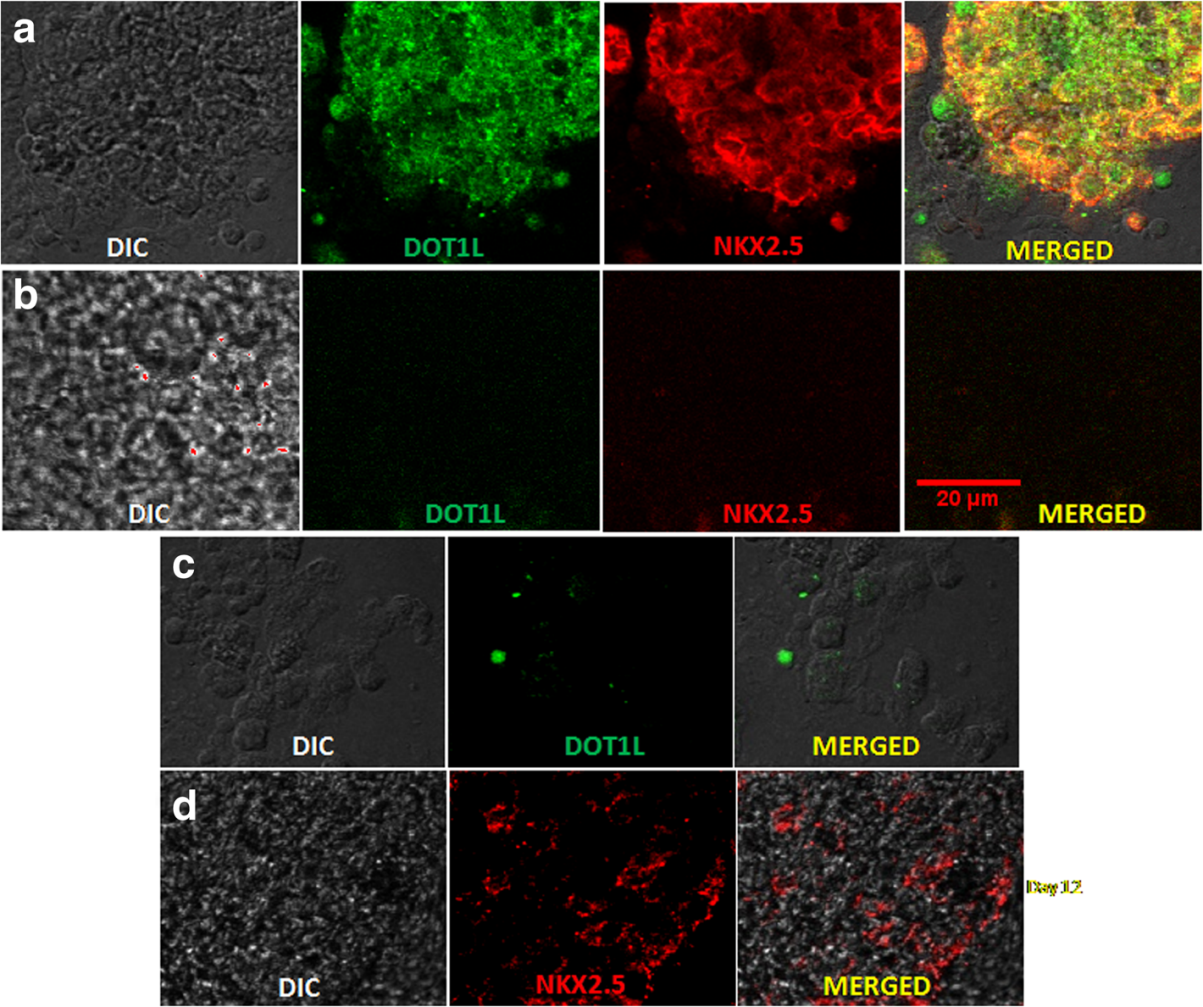
Effect of DOT1L knockdown on coexpression of DOT1L and NKX2.5 during KIND1 differentiation into the cardiac lineage. Immunostaining for DOT1L and NKX2.5 in KIND1 cells during cardiac differentiation. **a** Coexpression of DOT1L and NKX2.5 in nontarget siRNA-treated controls at day 12. **b** Coexpression of DOT1L and NKX2.5 in siRNA-treated cells on day 12. **c**, **d** Expression of DOT1L and NKX2.5 in siRNA-treated HES3 cells at days 0 and 12 respectively. Scale bar: 20 μm. DIC differential interference contrast

**Fig. 8:**
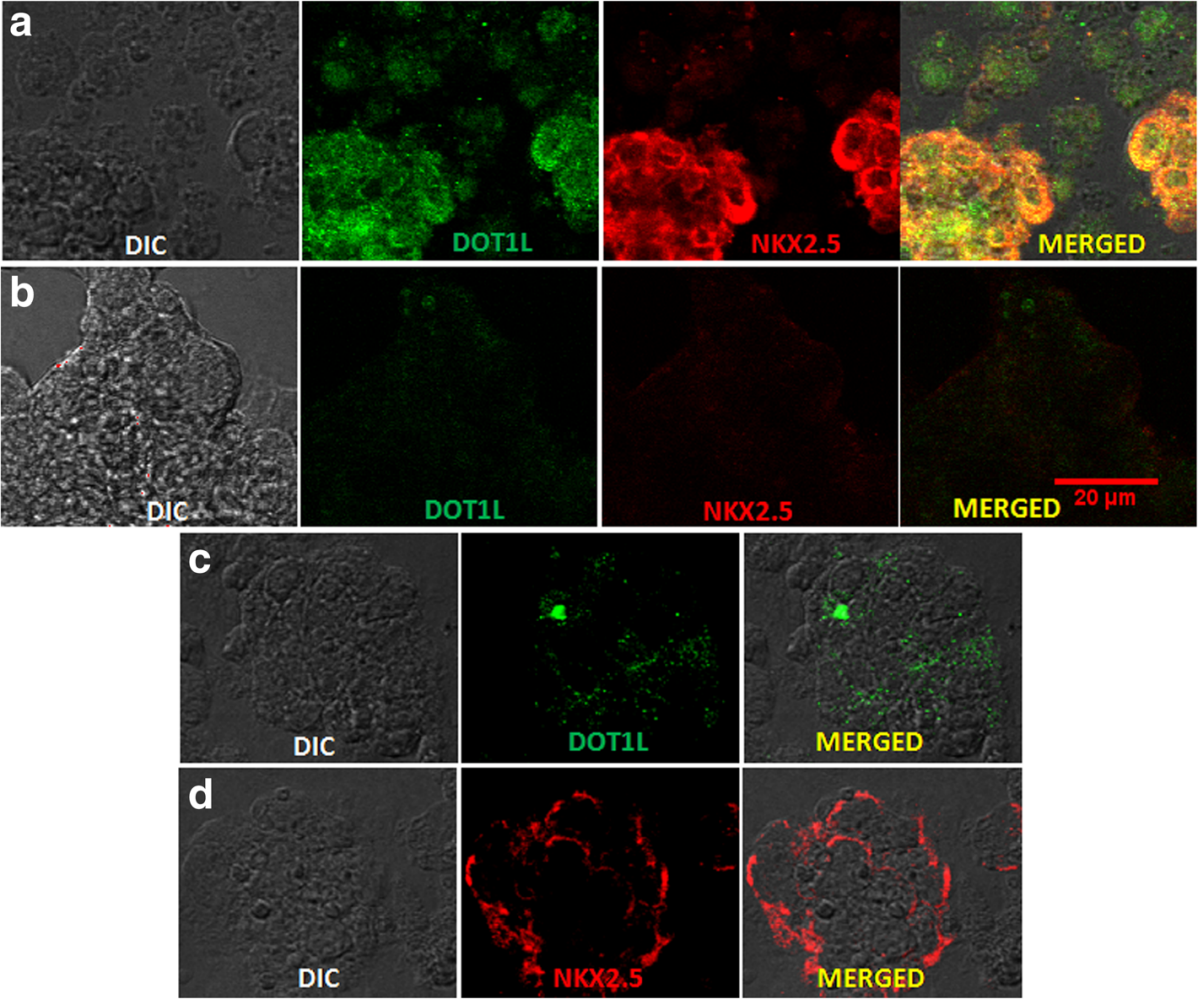
Effect of DOT1L knockdown on coexpression of DOT1L and NKX2.5 during HES3 cell differentiation into the cardiac lineage. Immunostaining for DOT1L and NKX2.5 in HES3 cells during cardiac differentiation. **a** Coexpression of DOT1L and NKX2.5 in nontarget siRNA-treated controls at day 12. **b** Coexpression of DOT1L and NKX2.5 in DOT1L siRNA-treated HES3 cells at days 12. **c**, **d** Expression of DOT1L and NKX2.5 in siRNA-treated HES3 cells at days 0 and 12 respectively. Scale bar: 20 μm. DIC differential interference contrast

Further qPCR analysis was carried out to understand the status of *NKX2.5 *expression at the transcript level (Fig. 6). While the expression of *NKX2.5* in progenitor cells containing no target siRNA was comparable to that obtained from wild-type cells, the levels of *NKX2.5 *expression in progenitors derived from cells deficient for DOT1L were drastically affected at both progenitor (day 12) and cardiomyocyte (day 20) stages when compared with control or wild-type cells at the respective stages. About 60% and 80% of reduced *NKX2.5* levels were denoted at day 12 and day 20 respectively, differentiated from both KIND1 and HES3 cells. This indicates that depletion of DOT1L in hES cells resulted in significantly reduced NKX2.5 expression and thus cardiac progenitor formation. To further investigate whether DOT1L is crucial for the downstream targets of *NKX2.5*, we checked for the expression of other cardiac representative genes like *MESP1*, *MEF2C*, *ISL1*, and *CTNT* in progenitors and cardiomyocytes obtained from KIND1 and HES3 cells. An intensely diminished gene expression level of these markers was noted at day 12 that was further reduced at day 20. These observations extend the effects of depletion of DOT1L according to which not only *NKX2.5* but also the downstream transcriptional targets of *NKX2.5* are influenced leading to much less or no cardiomyocyte formation *in vitro*. To further confirm these results, we performed dual immunofluorescence to study the coexpression of NKX2.5 and DOT1L in cardiac progenitors deficient for DOT1L (Figs. 7 and 8). While we observed considerable areas, displaying their colocalization in control and wild-type hES cells, there was very little or no expression of either markers in the cardiac progenitors containing siDOT1L. These results further help establish the fact that DOT1L has a decisive participation in early incidents of cardiac differentiation from hES cells.

## Discussion

In the present study, we aimed to understand the importance of active histone modifier DOT1L during cardiac differentiation *in vitro* on hES cells. In-house-derived KIND1 and HES3 cell lines were used for the study. The ability of KIND1 cells to differentiate into cardiac progenitors and beating cardiomyocytes has been published previously [7, 41]; however, the present study provides the first results on cardiac differentiation of HES3 cells using a directed differentiation protocol. ChIP sequencing was performed to look for the DOT1L specific mark H3K79me2 in differentiating KIND1 and HES3 cells. As per the known localization of H3K79me2 modification, significant peaks were noted at the downstream regions of pluripotent genes like *OCT4*, *SOX2* and *NANOG*, whereas genes like *GATA4*, *HAND1*, *NR2F2*, *NKX2.5*, *MESP1*, *ISL1*, and *WNT5A* harbored the H3K79me2 peaks as the cells underwent differentiation into cardiac progenitor and cardiomyocyte stages. ChIP sequencing analysis also revealed the significant peaks of H3K79me2 on the *DMD* gene at days 12 and 20 in both KIND1 and HES3 cells, suggesting its direct upregulation by DOT1L during cardiac differentiation. Employing dual immunofluorescence, colocalization of DOT1L was studied with the master cardiac transcription factor *NKX2.5*. While *NKX2.5* was not expressed in the pluripotent stage, substantial areas showing coexpression of DOT1L and NKX2.5 were located in cardiac progenitors upon differentiation from both KIND1 and HES3 cells. Moreover, expression of *DMD* was also increased as undifferentiated KIND1 hES cells differentiated into cardiac progenitors and cardiomyocytes, and correlated with the DOT1L expression and H3K79me2 methylation. Further studies were undertaken to study the effects of loss of DOT1L on differentiating KIND1 and HES3 cells. A 70–75% knock down of DOT1L was obtained in hES cells using siRNA technology. Remarkably, DOT1L knockdown did not show any deleterious effects on the pluripotency of hES cells maintaining their typical morphology as well as the expression of pluripotency gene *OCT4*; however, deficiency of DOT1L severely attenuated the cardiac differentiation pattern in KIND1 cells as well as HES3 cells. Furthermore, transcription factor *NKX2.5* and its downstream targets like *GATA4*, *TBX5*, and *ISL1* were found to be critically downregulated at cardiac progenitor and cardiomyocyte stages when DOT1L was knocked down. This was further confirmed when cells lacking DOT1L did not show coexpression of NKX2.5 and DOT1L by immunofluorescence. We report the possible involvement of histone activating methyltransferase DOT1L during cardiac differentiation of hES cells. Such studies, besides helping to improve the efficiency of hES cell differentiation, would further aid in better understanding the early events underlying cardiac differentiation *in vitro*.

High levels of active methylation marks occur upon the euchromatin or the open chromatin that is accessible to the transcription machinery. Cardiac cell fate also depends upon its specific and timely gene expression mechanisms that in turn are largely regulated by active epigenetic modifications like H3K4me3, H3K36me3, and H3K79me2. H3K4me3 is indispensable in cardiac developmental genes, evidenced by exome sequencing to identify the underlying mutations in CHD patients. This includes genes like *GATA4*, *NKX2.5*, and *TBX5* read with mutated H3K4 methylation [49]. Crucial involvement of MLL2 is also visualized in differentiating mES cells that directly controls cardiac specific genes by promoting H3K4me3 deposition [50]. H3K36me3 represents a second crucial active methylation mark found to be enriched upon NKX2.5. Interaction of H3K36me3 methyltransferase WHSC1 and NKX2.5 has remarkably shown the regulation of another set of genes like *PDGFRA* and *NPPA*. In support of this, WHSC1 mutant hearts resulted in cardiac developmental defects consistent with *NKX2.5* heterozygous mutants, further confirming their functional link [51]. Results of the present study show the key role of the transcription activating mark represented by H3K79me2 deposited onto the actively transcribing genes by DOT1L. These findings suggest the involvement of multiple histone activating methyltransferases for the activation of a gene in cardiac differentiation. However, what further remains to be revealed is whether there exists a collaborative effort by these epigenetic modifiers and also the effects of loss of one enzyme on the functions of another epigenetic modifier.

NKX2.5 represents a critical cardiac developmental factor that essentially directs the multiple downstream genes required for cardiac morphogenesis and maturation. This is supported by the conduction and contraction defects leading to premature death upon its deletion *in vivo* [52–54]. *NKX2.5* mutations also predispose the patients to cardiac developmental disorder termed dilated cardiomyopathy (DCM). Involvement of NKX2.5 in processes like cardiomyocyte specification and their homeostasis, development of the conduction system and cardiac muscle cells, as well as septation and nodal formation makes it a central participant in the genetic model for DCM [55–58]. Several reports also implicate epigenetic factors as causal events of DCM. Nguyen and Zhang [38] showed the significant role of DOT1L in the pathogenesis of DCM and that cardiac-specific conditional knockout for DOT1L in mice was lethal in nature. This study also reported mechanistic details showing DMD as the key target mediating DOT1L function in cardiac cells. Our results are in agreement with these published reports and provide a possible mechanism involving DOT1L during cardiogenesis leading to various pathologies. This further opens up the question of whether the genetic cause of DCM with respect to NKX2.5 is due to failure of its activation by DOT1L and hence its loss of function.

Our study opens up a number of areas that further need to be explored in order to design a DOT1L-centered gene expression model. Similarly, the other gene activating factors might also be involved along with DOT1L since DOT1L is known to function in coordination with MLL2 for maintenance of gene expression in leukemia [59]. Crosstalk among histone methyltransferases also requires further investigation. On the other hand, understanding the effects of overexpression of DOT1L during *in-vitro* cardiac differentiation might also uncover newer layers of regulation of cardiac gene expression.

## Conclusions

The present study, besides uncovering the contribution of DOT1L in cardiac differentiation from hES cells, puts forward a wide range of exciting possibilities that would aid in enhancing the efficiency of cardiac differentiation from hES cells as well as their clinical applications. However, further studies showing altered occupancy of H3K79me2 mark post DOT1L knock down as well as demonstrating direct binding of DOT1L to NKX2.5 in a pure population of cardiomyocytes need to be studied in order to further substantiate our findings.

## Additional files

Additional file 1: Brightfield images of HES3 hES cells during directed differentiation into cardiac lineage. Differentiation results in distinct morphological changes leading to increased compaction among the cells as differentiation proceeds from day 0 to day 20. Similar changes observed when KIND1 cells were differentiated into cardiac cells as described earlier [43]. Magnification 10×. (PDF 554 kb)

Additional file 2: Characterization of cardiac differentiation of HES3 cells by quantitative real-time PCR (qRT-PCR). Expression of transcripts representing pluripotency (*OCT4*), cardiac mesoderm (*MESP1*), cardiac progenitors (*NKX2.5*, *MEF2C*), and cardiomyocytes (*CTNT*) at days 0, 12, and 20 during 20 days of cardiac differentiation. Note *OCT-4* expression in undifferentiated cells is downregulated as the cells initiate differentiation. Early cardiac markers detected on day 12 and mature markers upregulated on day 20. Similar changes in transcripts expression observed when KIND1 cells were differentiated into cardiac cells as described earlier [43]. Error bars represent ±SEM. (PDF 410 kb)

Additional file 3: Characterization of cardiac differentiation of HES3 cells by immunofluorescence studies. Expression of NKX2.5 (**A**) and CTNT (**B**) on days 12 and 20 observed by immunofluorescence. (**A**) Distinct nuclear expression of NKX2.5 observed and (**B**) CTNT cell surface expression. Similar changes observed when KIND1 cells were differentiated into cardiac cells as described earlier [43]. Counterstaining using DAPI. Magnifications 20×. (PDF 450 kb)

Additional file 4: ChIP sequencing in KIND1 and HES3 cells during cardiac differentiation visualized by Integrated Genome Viewer shows binding profile of H3K79me2 modification across genes *HNF4A*, *LEFTY1*, *NOGGIN*, *NQO1*, *OTX2*, and *NPTX2* in KIND1 (green) and HES3 (red) cells at days 0, 12, and 20 of cardiac differentiation. (PDF 548 kb)

Additional file 5: Dystrophin gene expression during cardiac differentiation of KIND1 hES cells on days 0, 12, and 20 during cardiac differentiation of KIND1 hES cell line. Expression of Dystrophin increased in cardiac progenitors and cardiomyocytes compared to undifferentiated KIND1 cells. Results in agreement with earlier reports in DOT1L conditional knockout mice heart concluding Dystrophin as a direct target of DOT1L [35]. Error bars represent ±SEM. (PDF 329 kb)

Additional file 6: ChIP sequencing of occupancy of H3K79me2 on DMD gene during cardiac differentiation of KIND1 and HES3 cells showing occupancy of H3K79me2 methylation mark brought about by DOT1L on DMD gene during cardiac differentiation. Results clearly show significant peaks representing the DOT1L specific methylation mark on days 12 and 20 as compared to day 0 suggestive of its activation by DOT1L during cardiac differentiation *in vitro.* (PDF 614 kb)

## Abbreviations

bFGF: Basic fibroblast growth factor
CHD: Chronic heart disease
ChIP: Chromatin immunoprecipitation
hES: Human embryonic stem
PBS: Phosphate buffered saline
PcG: Polycomb group
PCR: Polymerase chain reaction
PSC: Pluripotent stem cell
TrxG: Trithorax group
