# Supplementary material for: Transcriptional activator DOT1L putatively regulates human embryonic stem cell differentiation into the cardiac lineage
Source: Stem Cell Res Ther. 2018 Apr 10;9:97. doi: 10.1186/s13287-018-0810-8 (PMC5891944; doi:10.1186/s13287-018-0810-8)
Supplement: Supplementary file 1 — Brightfield images of HES3 hES cells during directed differentiation into cardiac lineage. Differentiation results in distinct morphological changes leading to increased compaction among the cells as differentiation proceeds from day 0 to day 20. Similar changes observed when KIND1 cells were differentiated into cardiac cells as described earlier [43]. Magnification 10×. (PDF 554 kb) [file 13287_2018_810_MOESM1_ESM.pdf]

Additional File 1

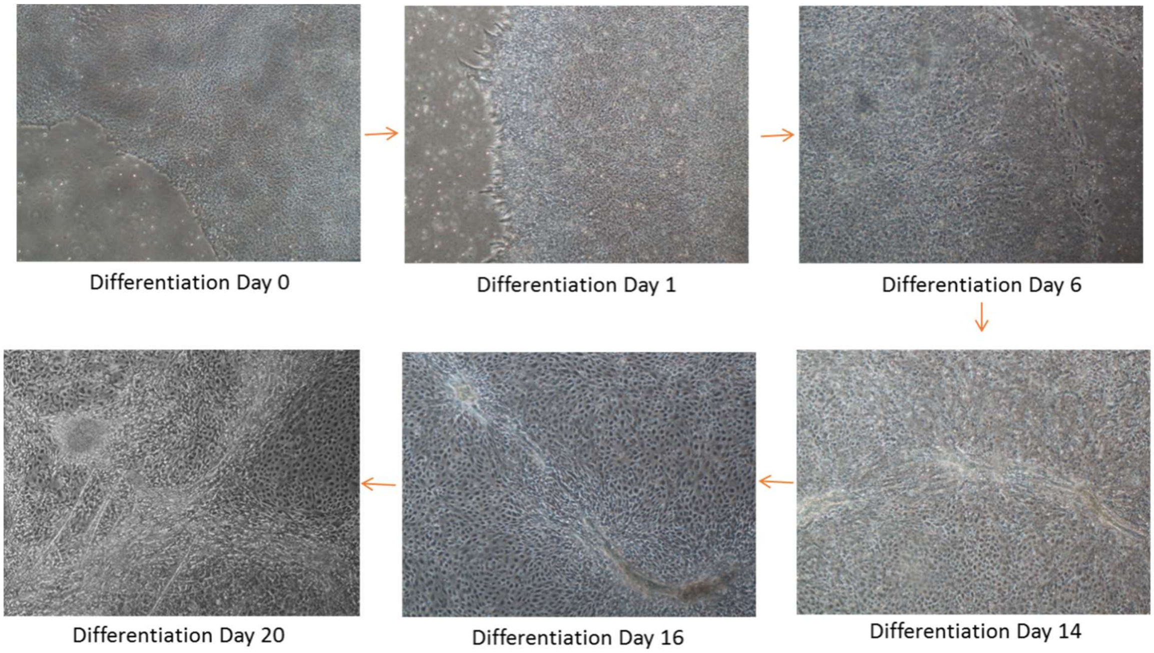

Morphological changes in HES3 cells as they undergo directed differentiation into the cardiac lineage. Bright field images of HES3 hES cells during their directed differentiation into cardiac lineage. Differentiation results in distinct morphological changes leading to increased compaction among the cells as differentiation proceeds from day 0 to day 20. Similar changes were observed when KIND1 cells were differentiated into cardiac cells as described earlier (Pursani et al, 2017). Magnification 10X
