# Supplementary material for: Transcriptional activator DOT1L putatively regulates human embryonic stem cell differentiation into the cardiac lineage
Source: Stem Cell Res Ther. 2018 Apr 10;9:97. doi: 10.1186/s13287-018-0810-8 (PMC5891944; doi:10.1186/s13287-018-0810-8)
Supplement: Supplementary file 2 — Characterization of cardiac differentiation of HES3 cells by quantitative real-time PCR (qRT-PCR). Expression of transcripts representing pluripotency (OCT4), cardiac mesoderm (MESP1), cardiac progenitors (NKX2.5, MEF2C), and cardiomyocytes (CTNT) at days 0, 12, and 20 during 20 days of cardiac differentiation. Note OCT-4 expression in undifferentiated cells is downregulated as the cells initiate differentiation. Early cardiac markers detected on day 12 and mature markers upregulated on day 20. Similar changes in transcripts expression observed when KIND1 cells were differentiated into cardiac cells as described earlier [43]. Error bars represent ±SEM. (PDF 410 kb) [file 13287_2018_810_MOESM2_ESM.pdf]

Additional File 2

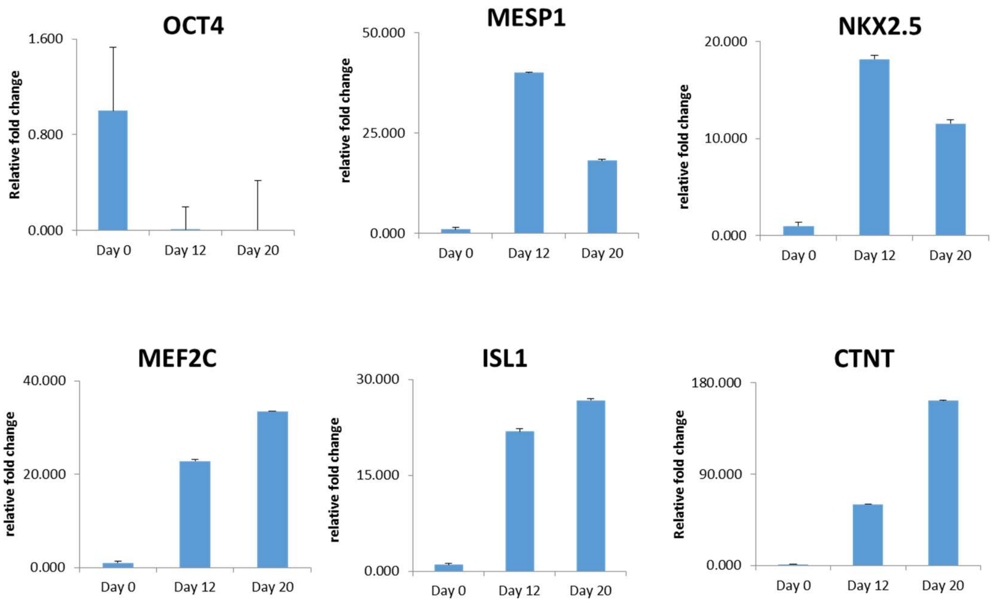

Characterization of cardiac differentiation of HES3 cells by quantitative real-time PCR (qRT-PCR). Expression of transcripts representing pluripotency (OCT4), cardiac mesoderm (MESP1), cardiac progenitors (NKX2.5, MEF2C) and cardiomyocytes (CTNT) at days 0, 12 and 20 during 20 days cardiac differentiation. Note OCT-4 expression in undifferentiated cells which is down-regulated as the cells initiate differentiation. Early cardiac markers are detected on Day 12 and mature markers are up-regulated on Day 20. Similar changes in transcripts expression was observed when KIND1 cells were differentiated into cardiac cells as described earlier (Pursani et al, 2017). Error bars represent ± SEM.
