# Supplementary material for: Transcriptional activator DOT1L putatively regulates human embryonic stem cell differentiation into the cardiac lineage
Source: Stem Cell Res Ther. 2018 Apr 10;9:97. doi: 10.1186/s13287-018-0810-8 (PMC5891944; doi:10.1186/s13287-018-0810-8)
Supplement: Supplementary file 3 — Characterization of cardiac differentiation of HES3 cells by immunofluorescence studies. Expression of NKX2.5 (A) and CTNT (B) on days 12 and 20 observed by immunofluorescence. (A) Distinct nuclear expression of NKX2.5 observed and (B) CTNT cell surface expression. Similar changes observed when KIND1 cells were differentiated into cardiac cells as described earlier [43]. Counterstaining using DAPI. Magnifications 20×. (PDF 450 kb) [file 13287_2018_810_MOESM3_ESM.pdf]

Additional File 3

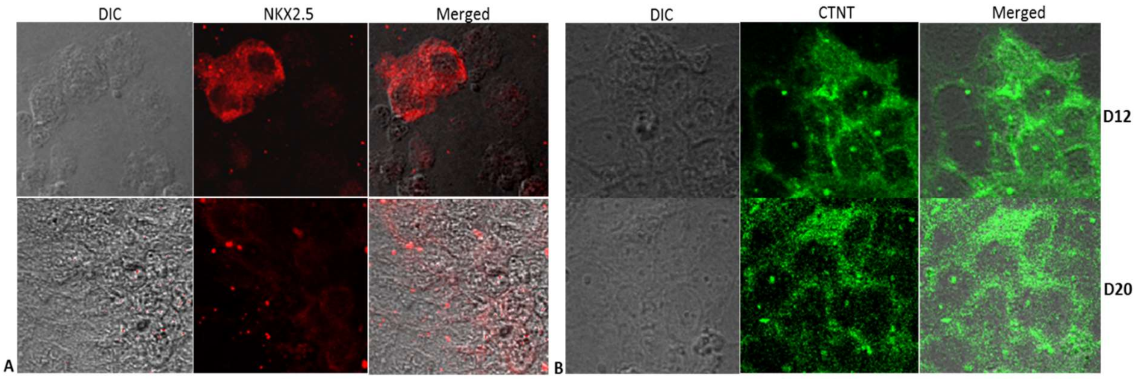

Characterization of cardiac differentiation of HES3 cells by immuno-fluorescence studies. Expression of NKX2.5 (A) and CTNT (B) on days 12 and 20 observed by immuno fluorescence. (A) Distinct nuclear expression of NKX2.5 is observed and (B) CTNT show cell surface expression. Similar changes were observed when KIND1 cells were differentiated into cardiac cells as described earlier (Pursani et al, 2017). Counter staining was done using DAPI. Magnifications 20X.
