# Supplementary material for: Transcriptional activator DOT1L putatively regulates human embryonic stem cell differentiation into the cardiac lineage
Source: Stem Cell Res Ther. 2018 Apr 10;9:97. doi: 10.1186/s13287-018-0810-8 (PMC5891944; doi:10.1186/s13287-018-0810-8)
Supplement: Supplementary file 4 — ChIP sequencing in KIND1 and HES3 cells during cardiac differentiation visualized by Integrated Genome Viewer shows binding profile of H3K79me2 modification across genes HNF4A, LEFTY1, NOGGIN, NQO1, OTX2, and NPTX2 in KIND1 (green) and HES3 (red) cells at days 0, 12, and 20 of cardiac differentiation. (PDF 548 kb) [file 13287_2018_810_MOESM4_ESM.pdf]

Additional File 4

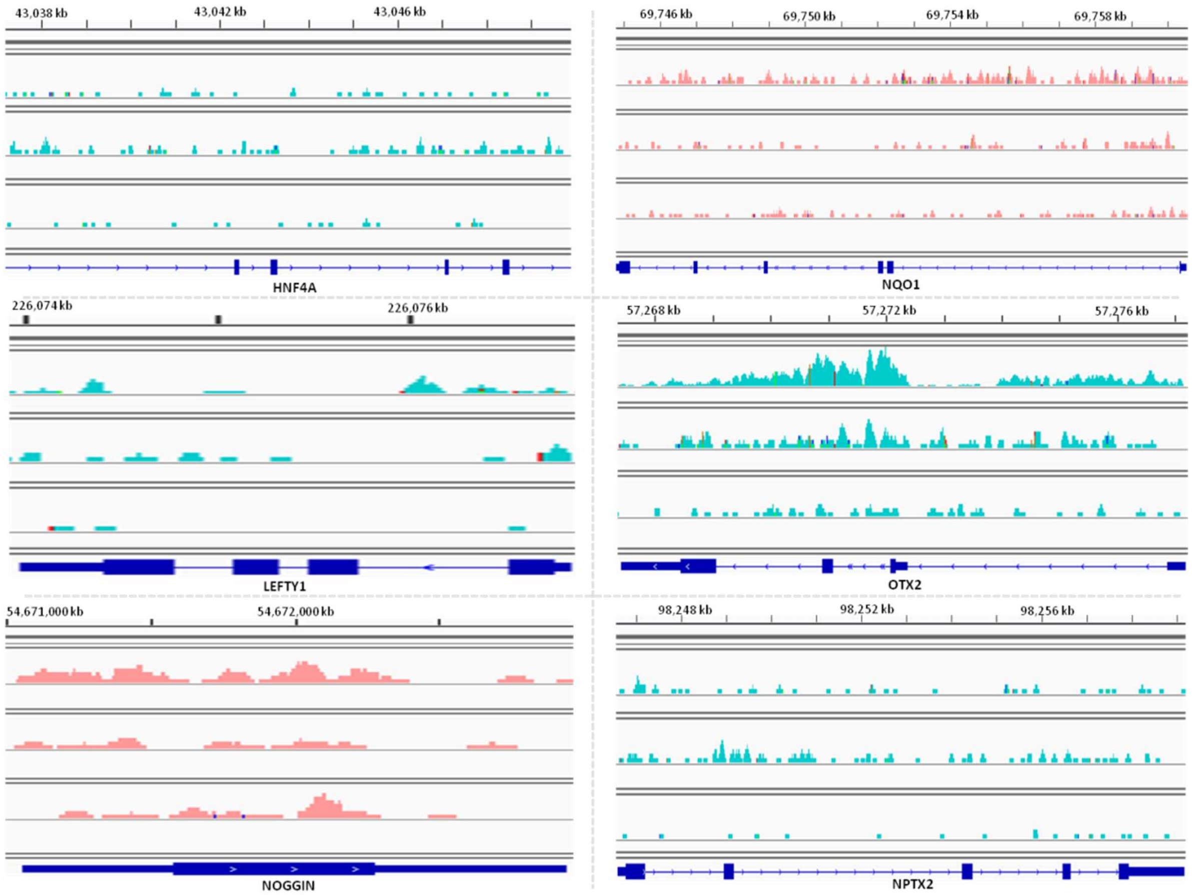

ChIP-sequencing in KIND1 & HES3 during cardiac differentiation. Analysis of ChIP- sequencing visualized by integrated genome viewer shows the binding profile of H3K79me2 modification across the genes of HNF4A, LEFTY1, NOGGIN, NQO1, OTX2, NPTX2 in KIND1 (green) and HES3 (red) cells at days 0, 12 and 20 of cardiac differentiation.
