# Supplementary material for: Transcriptional activator DOT1L putatively regulates human embryonic stem cell differentiation into the cardiac lineage
Source: Stem Cell Res Ther. 2018 Apr 10;9:97. doi: 10.1186/s13287-018-0810-8 (PMC5891944; doi:10.1186/s13287-018-0810-8)
Supplement: Supplementary file 5 — Dystrophin gene expression during cardiac differentiation of KIND1 hES cells on days 0, 12, and 20 during cardiac differentiation of KIND1 hES cell line. Expression of Dystrophin increased in cardiac progenitors and cardiomyocytes compared to undifferentiated KIND1 cells. Results in agreement with earlier reports in DOT1L conditional knockout mice heart concluding Dystrophin as a direct target of DOT1L [35]. Error bars represent ±SEM. (PDF 329 kb) [file 13287_2018_810_MOESM5_ESM.pdf]

Additional Figure 5

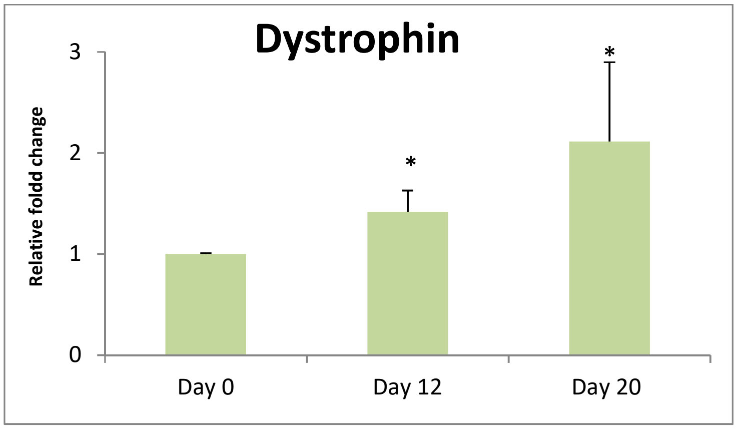

Dystrophin gene expression during cardiac differentiation of KIND1 hES cells. qRT-PCR analysis for Dystrophin gene on days 0, 12 and 20 during cardiac differentiation of KIND1 hES cell line. Expression of Dystrophin is increased in cardiac progenitors and cardiomyocytes compared to undifferentiated KIND1 cells. The results are in agreement with the earlier reports in DOT1L conditional knockout mice heart concluding the Dystrophin as a direct target of DOT1L (Nguyen et al. 2011). Error bars represent ± SEM.
