# Supplementary material for: Transcriptional activator DOT1L putatively regulates human embryonic stem cell differentiation into the cardiac lineage
Source: Stem Cell Res Ther. 2018 Apr 10;9:97. doi: 10.1186/s13287-018-0810-8 (PMC5891944; doi:10.1186/s13287-018-0810-8)
Supplement: Supplementary file 6 — ChIP sequencing of occupancy of H3K79me2 on DMD gene during cardiac differentiation of KIND1 and HES3 cells showing occupancy of H3K79me2 methylation mark brought about by DOT1L on DMD gene during cardiac differentiation. Results clearly show significant peaks representing the DOT1L specific methylation mark on days 12 and 20 as compared to day 0 suggestive of its activation by DOT1L during cardiac differentiation in vitro. (PDF 614 kb) [file 13287_2018_810_MOESM6_ESM.pdf]

Additional File 6

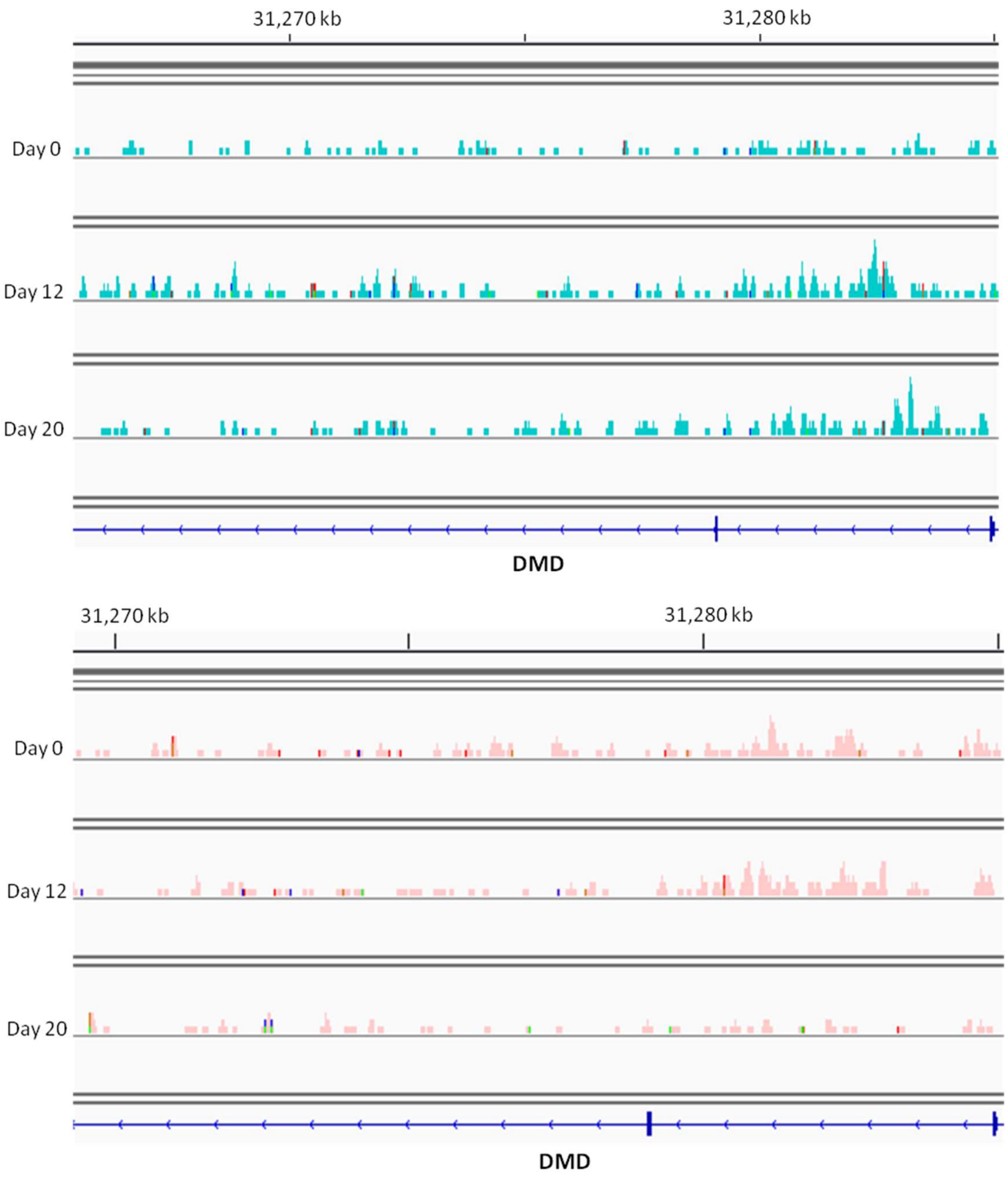

ChIP sequencing showing the occupancy of H3K79me2 on the DMD gene during cardiac differentiation of KIND1 and HES3. Analysis of the ChIP sequencing data showing the occupancy of H3K79me2 methylation mark brought about by DOT1L on the DMD gene during cardiac differentiation. The results clearly show the significant peaks representing the DOT1L specific methylation mark on days 12 and 20 as compared to day 0 suggestive of its activation by DOT1L during cardiac differentiation *in vitro*.
